# Pantoprazole-Induced Bone Loss through Gastrin Secretion: A Stereological Study

**DOI:** 10.1155/2023/2594664

**Published:** 2023-09-06

**Authors:** Forough Saki, Mesbah Shams, Sanaz Dastghaib, Farhad Koohpeyma

**Affiliations:** Endocrinology and Metabolism Research Center, Shiraz University of Medical Sciences, Shiraz, Iran

## Abstract

**Background:**

Recent researches have failed to uncover a clear explanation for proton pump inhibitors' bone-loss effects. In light of pantoprazole's effects on gastrin secretion, the goal of this study was to see if it caused bone loss through gastrin secretion.

**Methods:**

Forty male rats were divided into control, octreotide (Oct), pantoprazole (Pan), and pantoprazole plus octreotide (Pan+Oct) groups. Serum calcium, phosphorous, alkaline phosphatase, parathyroid hormone, and gastrin were measured before and three months after the treatment, and bone densitometry was examined. The rats' femoral bones were examined stereologically at the end of the investigation.

**Results:**

The Pan group had considerably greater levels of serum alkaline phosphatase, parathyroid hormone (PTH), and gastrin, but this was prevented in the presence of Oct, a gastrin secretion inhibitor. All parameters of femoral bone densitometry in the Pan group were significantly lower than the control after treatment which was considerably inhibited in the presence of Oct. Furthermore, when compared to the control and Oct groups, the rats in the Pan group had a lower trabecular volume, femur bone weight, and volume, as well lower number of osteocytes. The amount of osteoclasts, on the other hand, was much higher in the Pan group than in the other groups.

**Conclusion:**

Overall findings revealed that pantoprazole caused bone loss, which could be prevented by adding octreotide. Because these detrimental effects were not detected in rats given both Oct and Pan, it was suggested that the effect of Pan on bone was produced by a hypergastrinemic condition.

## 1. Introduction

Proton pump inhibitors (PPIs) are among the most widely prescribed drug classes worldwide [1]. They are potent gastric acid-suppressing drugs used to prevent or treat peptic ulcers, gastroesophageal reflux disease (GERD), and erosive esophagitis [2]. In spite of the evident clinical benefits of PPIs, recent epidemiological studies have raised concerns about their side effects after long-term use [3, 4]. PPIs are commonly well tolerated; however, some previous studies showed an association between PPIs and hypomagnesemia, pneumonia, pseudomembranous colitis, pancreatic cancer, and dementia [5–9]. There have also been some recent concerns about the effects of PPIs on bone metabolism including fractures, osteoporosis, and low bone mass [10]. Although numerous epidemiological studies have reported that treatment using PPIs reduced bone mineral density (BMD) [1, 11–13], others have failed to find a significant association [14–17]. Moreover, three systematic reviews and meta-analyses failed to find a definite mechanism for the effect of PPIs on bones [18–20] and suggested the need for observational and randomized controlled trials to resolve the conflict. Although the mechanism of the effect of PPIs on bones has remained unclear, several plausible explanations have been suggested including a decrease in calcium absorption from the intestine [21], a reduction in vitamin B12 absorption [22], and secondary hyperparathyroidism induced by secondary hypergastrinemia [23, 24]. One possible hypothesis is that the acidosis induced by this class of drugs increases the secretion of parathyroid hormone (PTH), followed by the release of calcium from the bones to restore pH balance. However, the details have remained unclear in the recent reviews evaluating the effects of PPIs on BMD [1, 10, 18]. One study indicated that long-term PPI therapy induced moderate hypergastrinemia in most patients and increased the prevalence of enterochromaffin-like (ECL) cell hyperplasia [25]. The fact that stomach acid blockage causes higher levels of gastrin is one reason for concern about the long-term use of PPIs. The G cells of the gastric antrum have a homoeostatic reaction to the lowered acidity of the gastric juice [26]. Gastrin has been demonstrated to exert trophic effects on tissues throughout the gastrointestinal tract including ECL cells in the oxyntic mucosa [27]. In a previous study, hypergastrinemia caused by the lifelong treatment of high doses of PPIs or histamine H2 receptor antagonists [28, 29] or partial gastric corpectomy was linked to ECL cell hyperplasia and neuroendocrine tumors (NETs) in female rats [25]. Short-term injection of gastrin transiently increased Ca absorption by the bone [15], whereas long-term exogenously generated hypergastrinemia decreased bone mass while enhancing bone turnover [16]. It was previously thought that gastrin's hypocalcemic activity was mediated by the gastrointestinal peptide gastrocalcin, which increased calcium uptake in the bones [30]. Gastrin has been shown to have a direct effect on calcium uptake in bones [31]. The concept that the stomach is vital for calcium homeostasis has been bolstered by new research works. Not only is the stomach crucial for secreting acid that aids in intestinal calcium absorption [31], but gastrin from the antrum has also been found to lower the blood level of calcium and may be responsible for immobilization-induced hypocalcaemia in rats [32, 33].

It has been reported that gastrectomy lowers bone weight, density, and mineral content [34] as well as the possibility of metabolic bone disease [35]. This implies that gastrin may play a role in bone metabolism. Firstly, gastrin prevents postprandial hypercalcemia by directing calcium from the bloodstream to bones and muscles through an intense and transitory action. On the other hand, a prior study indicated a significant increase in the circulation amount of gastrin during a meal [36, 37] and after an oral dosage of calcium carbonate in humans, suggesting the physiological effect of gastrin [38]. Secondly, by increasing calcium levels in the bone fluid compartment, gastrin may indirectly improve bone mineralization and turnover when bone matrix formation is normal but bone mineralization is impaired due to a calcium deficiency (e.g., during growth in vitamin D-deficient individuals). This long-term effect may be crucial for bone remodeling, particularly during growth and development [31, 33]. Considering PPIs' inhibitory effect on gastrin production and the fact that hypergastrinemia is a major cause of PPI-induced bone loss [39, 40], long-term usage of pump inhibitors can lead to osteoporosis. Oversecretion of gastrin and hypochlorhydria are two probable explanations. Octreotide is a somatostatin analogue that prevents the release of a variety of hormones including gastrin.

Up to now, the short-term effects of PPIs on bone have been well studied, but the consequences of long-term profound acid inhibition are not fully known [33]. Therefore, the present study is aimed at evaluating the changes in femoral BMD after the administration of pantoprazole to rats. In another group of rats, octreotide acetate was added to pantoprazole for inhibition of gastrin secretion in order to evaluate the effects of pantoprazole, independent of gastrin effects, on bone stereology and femoral BMD.

## 2. Materials and Methods

This experimental study was conducted on 40 adult male Sprague-Dawley rats (6 months old) weighing 250 ± 20 gr that were purchased from the animal laboratory of Shiraz University of Medical Sciences. The animals were housed in standard cages (four in each cage) at 22 ± 2°C with 12 : 12 hr light-dark cycles, were fed with a normal standard rodent chow diet, and had free access to tap water. All the rats underwent one-week acclimatization to the animal laboratory circumstances before the study. Then, the rats were divided into four groups of 10, as follows:

1. Control group received a standard rat chow diet and 1 cc of normal saline as the placebo
2. Octreotide group (Oct) received three doses of intramuscular octreotide 1 mg/per rat monthly (Novartis Pharma AG, Basle, Switzerland)
3. Pantoprazole group (Pan) received 3 mg/kg pantoprazole (Avicenna Company, Tehran, Iran) daily for three months based on a previous report [41]
4. Pantoprazole plus octreotide group (Pan+Oct) received 3 mg/kg of pantoprazole daily for three months and three doses of intramuscular injection of octreotide per month (1 mg/month)

After the three-month treatment, the rats' weight, biochemical analysis, and BMD were evaluated. At the end of the study, the rats were killed by thiopental overdose (100 mg/kg) under ketamine-xylazine (Alfasan, Netherlands) anesthesia. Samples for stereological studies were obtained from the cut edge of the left femoral bone. In addition, blood samples (5 mL) were collected via cardiac puncture and were centrifuged at 3500 rpm for 12 minutes before being stored at -70°C for further analysis of biochemical parameters, namely calcium, phosphate, alkaline phosphatase (ALP), parathyroid, and gastrin hormones.

This study was approved by the local Ethics Committee and vice-chancellor for Research Affairs of Shiraz University of Medical Sciences (IR.SUMS.MED.REC1399.245). The study was conducted in accordance with the Animal Research Reporting of In Vivo Experiment (ARRIVE) guidelines [42] and all the recent applicable institutional and national guidelines for animal care and use.

### 2.1. Biochemical Study and Bone Mineral Density

Serum calcium (mg/dL), phosphate (mg/dL), and ALP (IU/L) were measured through enzymatic colorimetric assays using a DIRUI (CS-T240, China) autoclinical chemistry analyzer and commercial diagnostic kits (Biosystem Company, Spain) before and at the end of the study. Additionally, serum levels of PTH (pg/mL) and gastrin (ng/L) were measured by ELISA using Bioassay Technology Laboratory kits (China). The coefficient of variation (CV) was less than 10%, and intra- and interassay CVs were <6% and <7%, respectively. Moreover, BMD was evaluated by a Hologic system Dual-energy X-ray absorptiometry (DXA) (Discovery W (S/N 84107), USA) with software for small animals at Shiraz Endocrinology and Metabolism Research Center. Based on the measurements in ten rats, the CV was 0.5% for the lumbar spine and 2.5% for the femur. Bone mineral content (BMC) was measured, as well. Besides, bone mineral apparent density (BMAD) was calculated through the following formula [43]:

(1) $$\begin{array}{c} \text{BMAD}=\frac{\text{BMC}}{{\text{area}}^{2}}. \end{array}$$

### 2.2. Stereology Study

**Figure 1:**
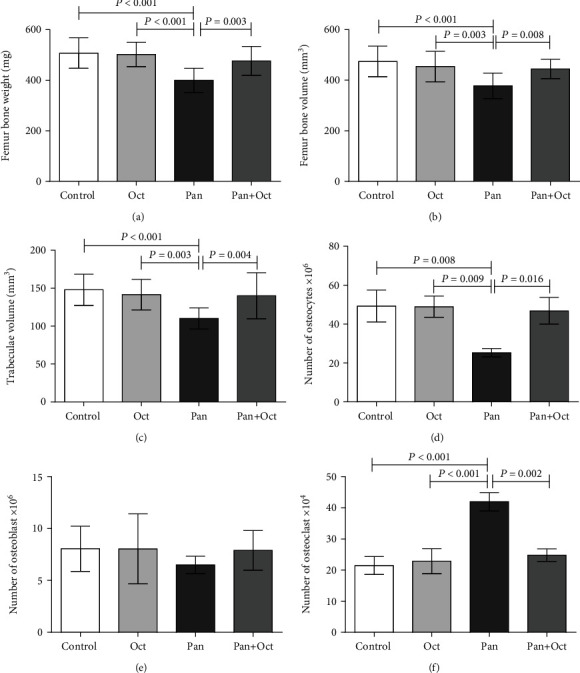
Statistical analysis of the stereological parameters in all the study groups at the end of the study. The data have been presented as mean ± SD; *p* < 0.05 was considered statistically significant. One-way ANOVA followed by LSD post hoc test was utilized. Con: control; Oct: octreotide; Pan: pantoprazole.

The primary volume of the femoral bone was measured using the immersion method [44]. Then, the bone sections were prepared according to the “orientate method” [45] adopted from a previous stereological study [46]. After staining the tissue sections with H&E (Figure 1), the degree of shrinkage was calculated using the following formula:

(2) $$\begin{array}{c} \text{Degree of shrinkage}=1-{\left(\frac{\text{Area after}}{\text{Area before}}\right)}^{1.5}. \end{array}$$

Additionally, the total volume of the femoral bone was calculated via the following formula:

(3) $$\begin{array}{c} \text{Final volume}=\left(1-\text{degree of shrinkage}\right)\times {\text{V}}_{\left(\text{primary}\right)}. \end{array}$$

A video-microscopy system including a Nikon microscope (E-200, Japan), a Samsung digital camera (SCB-2000 P, Korea), and a personal computer was used. Then, the trabecular volume was calculated by the Delesse formula [46–48] on 4 *μ*m-thick sections:

(4) $$\begin{array}{c} \text{Vv}\left(\text{trabecular}\right)=\frac{\sum_{i=1}^{n}p\;\left(\text{trabecular}\right)}{\sum_{i=1}^{n}p\;\left(\text{reference tissue}\right)},\\ \text{Trabecular volume}=\text{Vv}\left(\text{trabecular}\right)\times \text{Final volume}. \end{array}$$

The numerical density and absolute number of bone cells were estimated using the dissector method according to a standard protocol obtained from the research carried out by Noorafshan et al. [46]. The numerical density of the cells was calculated through the following formula:

(5) $$\begin{array}{c} \text{Nv}=\frac{\sum_{i=1}^{n}Q}{\sum_{i=1}^{n}P\times h\times \left(a/f\right)}\times \frac{t}{\text{BA}}, \end{array}$$

where *ΣQ* is the number of the whole cells counted in all the dissectors, *h* is the height of the optical dissector, *a*/frame is the area of the counting frame, *ΣP* is the total number of the counted frames, BA is the microtome block advance to cut the block, and *t* is the mean of the final section thickness.

The total number of bone cells was estimated using the following formula:

(6) $$\begin{array}{c} N\left(\text{bone cells}\right)=N_{v}\times V_{\text{Final}}. \end{array}$$

### 2.3. Statistical Analysis

All statistical analyses were done using the SPSS software (version 23; SPSS Ins, Chicago, USA). At first, the Kolmogorov-Smirnov test was used to determine the normality of the data. Due to the normality of the data and homogeneity of variances, parametric tests were used. A one-way ANOVA followed by LSD post hoc test was used to assess the significant differences between the means of the variables in four independent groups. Additionally, a paired sample *t*-test was used to evaluate the differences in each group before and after the study. *p* values less than 0.05 were considered statistically significant.

## 3. Results

### 3.1. The Effects of Long-Term Pantoprazole-Octreotide Consumption on Biochemical Parameters

**Figure 2:**
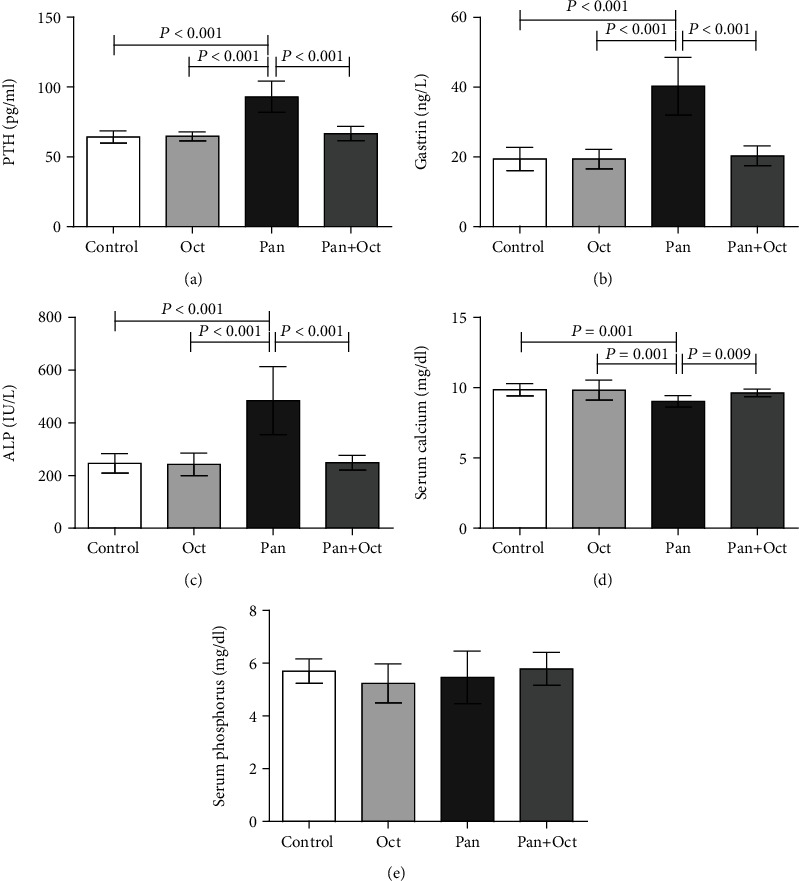
Comparison of the study groups regarding the serum levels of calcium, phosphorous, PTH, alkaline phosphatase, and gastrin before and at the end of the study. The data have been presented as mean ± SD. *p* < 0.05 was considered statistically significant. One-way ANOVA followed by LSD post hoc was used. Paired sample *t*-test was also used to analyze the data in each group. There are no significant differences between the columns containing at least one similar letter. However, different letters represent a significant difference. Con: control; Oct: octreotide; Pan: pantoprazole.

A comparison of the serum levels of calcium, phosphorous, and ALP before starting the treatment is summarized in Table 1. According to our baseline results, there were no significant differences observed between experimental groups in these parameters. As depicted in Figure 2, no significant changes were seen in the serum level of phosphorous in any of the study groups after 3 months of treatment. However, the amount of ALP increased significantly in Pan-treated rats compared to the control, Oct, and Pan+Oct groups (*p* < 0.001). On the other hand, at the end of 12 weeks, pantoprazole caused a significant decline in Ca level in comparison with other groups. It is worth mentioning that octreotide significantly prevented the effect of pantoprazole on the serum levels of calcium and ALP at the end of the study.

### 3.2. The Effects of Long-Term Pantoprazole-Octreotide Consumption on PTH and Gastrin Hormone

According to Table 1, no significant changes were observed in the serum levels of PTH and gastrin in all groups at the baseline. As demonstrated in Figure 2, there were no significant changes in the serum levels of PTH and gastrin in the control, Oct, and Pan+Oct groups at the end of the study. However, a significant increase was detected in the levels of PTH and gastrin in the Pan group (*p* < 0.001). It is worth noting that after the three-month treatment, octreotide could change the effect of pantoprazole and reduce the serum levels of PTH and gastrin without detectable differences in the control group. Our data showed no considerable differences in PTH and gastrin levels between the Pan+Oct and Oct groups.

### 3.3. The Effects of Long-Term Pantoprazole-Octreotide Consumption on the Femoral Bone Densitometry Parameters

**Figure 3:**
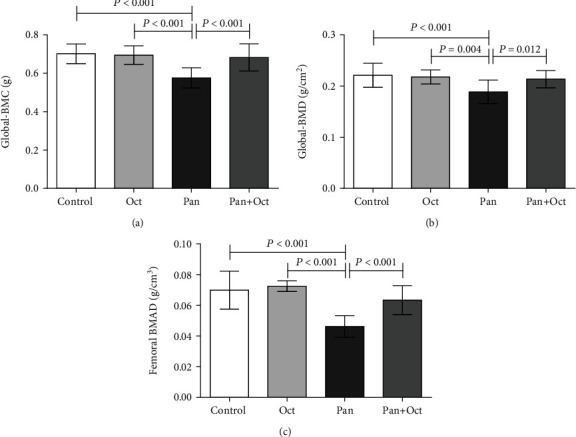
Femoral bone densitometry parameters in the four study groups after the different treatments. The data have been presented as mean ± SD; *p* < 0.05 was considered statistically significant. One-way ANOVA followed by LSD post hoc test was employed. Paired sample *t*-test was also used to analyze the data in each group. There are no significant differences between the columns containing at least one similar letter. However, different letters show a significant difference. Con: control; Oct: octreotide; Pan: pantoprazole; BMC: bone mineral content; BMD: bone mineral density; BMAD: bone mineral apparent density.

Table 2 shows the results of the femoral bone densitometry analysis (femoral BMC, BMD, and BMAD) before stating the treatment. There were no significant differences observed at the baseline level in bone densitometry parameters between the study groups. The results of femoral bone densitometry analysis at the end of 3 months of treatment are presented in Figure 3. The results showed a significant decrease in femoral BMC and BMD in the pantoprazole-treated rats compared to the control group at the end of the study (*p* < 0.001). Femoral BMAD, as a more accurate criterion of bone densitometry, also decreased in the pantoprazole group compared to the control group (*p* < 0.001). It is important to note that octreotide could prevent the effect of pantoprazole and increase femoral BMC, BMD, and BMAD in the Pan+Oct group at the end of the study (*p* < 0.001, *p* = 0.012, and *p* < 0.001, respectively).

### 3.4. The Effects of Long-Term Pantoprazole-Octreotide Consumption on Bone Stereological Parameters

The results of the stereological study are presented in Figure 1. The results indicated a significant decrease in femoral bone weight and volume in the Pan group compared to the control group at the end of the study (*p* < 0.001). The results also demonstrated that the rats in the pantoprazole group had a lower trabecular volume and a smaller number of osteocytes compared to the control group at the end of the study (*p* < 0.001 and *p* = 0.008, respectively). However, the number of osteoclasts was significantly higher in the pantoprazole-treated rats compared to the control group (*p* < 0.001) and the Pan+Oct group (*p* = 0.002). Moreover, no significant changes were observed in bone stereological parameters in the Pan+Oct group in comparison with the control group. It should be noted that octreotide could significantly inhibit the adverse effects of Pan on the femoral bone volume and weight, trabecular volume, and number of osteocytes and osteoclasts in the Pan+Oct group at the end of the study.

### 3.5. The Effects of Long-Term Pantoprazole-Octreotide Consumption on Bone Histopathological Changes

**Figure 4:**
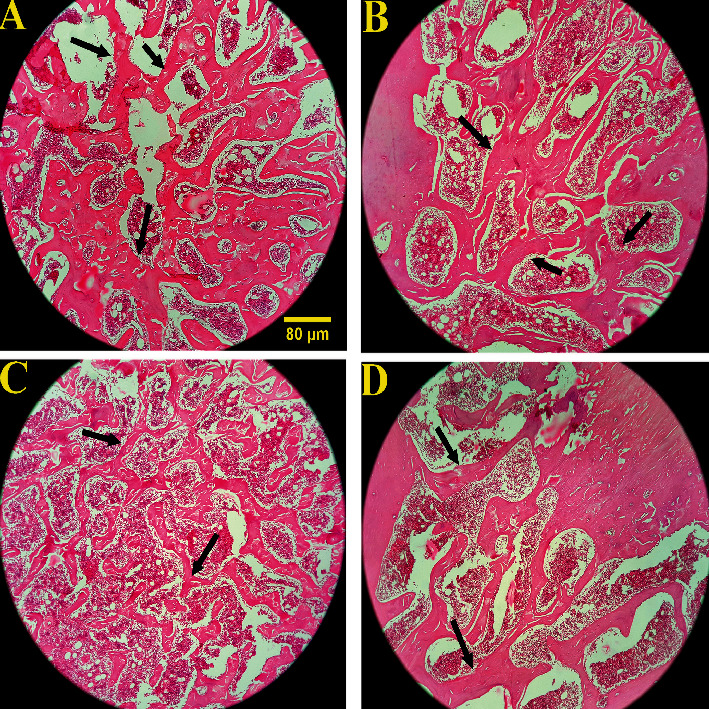
Optical photomicrograph of the changes in the trabecular bone tissue in the femoral head in the experimental groups (hematoxylin and eosin staining at ×100 magnification). (a) Healthy control rats with normal histoarchitecture in the trabecular bone. (b) Oct (octreotide) group: there were no pathological lesions in this group. (c) Pan (pantoprazole) group: severely thinned trabeculae. The results revealed a significant decrease in the trabecular volume in this group. (d) Pan+Oct (pantoprazole plus octreotide) group: there were no significant differences in the trabecular volume in this group compared to the control group. The arrows indicate trabecular thickness.

**Figure 5:**
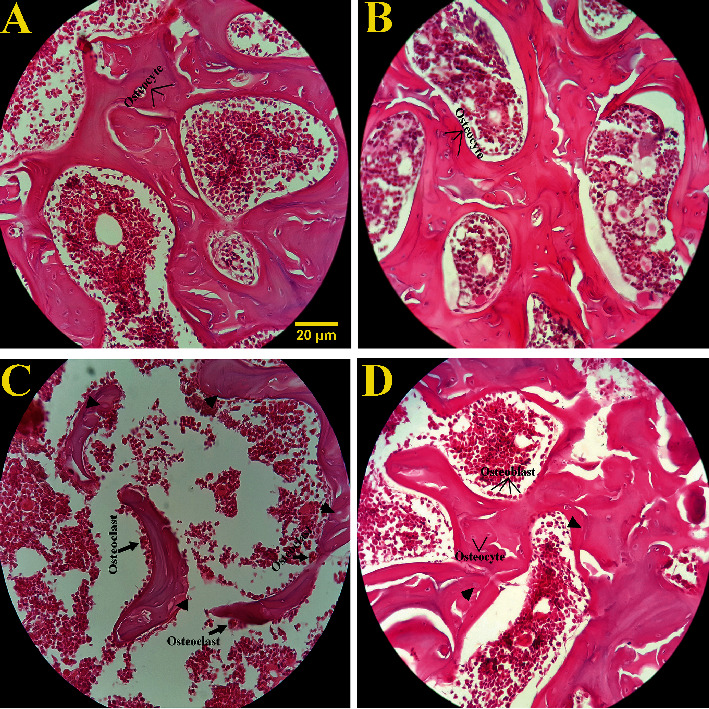
Qualitative analysis of the osteocytes, osteoblasts, and osteoclast cells of the femur tissue in the experimental groups (hematoxylin and eosin staining with ×400 magnification). (a) Healthy control group, (b) octreotide group, (c) Pan group, and (d) Pan+Oct group. The results showed a significant decrease in the number of osteocytes and bone lacuna depletion (pick arrow) in the Pan group compared to the control and Oct groups. A significant increase was also observed in the number of osteoclast cells in the Pan group compared to the other groups. However, no significant changes were detected in the number of osteoblast cells in the experimental groups.

The microscopic views of the bone tissues at two different magnifications are shown in Figures 4 and 5. The optical photomicrograph (×100) of the femoral head in the rats is presented in Figure 4. The results revealed severely thinned trabeculae in the Pan group. However, no significant change was observed in trabeculae in the Pan+Oct and Oct groups. The qualitative analysis of the osteocytes, osteoblasts, and osteoclasts of the femoral bone in the H&E staining of the femoral bone with ×400 magnification is depicted in Figure 5. The results showed a significant decrease in the number of osteocytes and bone lacuna depletion as well as a significant increase in the number of osteoclasts in the pantoprazole-treated group. Overall, octreotide could prevent the adverse bone histopathological changes induced by pantoprazole during three months.

## 4. Discussion

The present findings indicated that pantoprazole (Pan) decreased femoral bone density and femoral BMAD. Additionally, pantoprazole increased the serum levels of PTH, gastrin, and ALP and decreased the Ca level after three months of treatment. Besides, a decrease was found in the femoral bone weight and volume as well as the trabecular volume at the end of the study. Based on the results of the stereological study, pantoprazole decreased the number of osteocytes and increased the number of osteoclasts. Nonetheless, adding octreotide (gastrin secretion inhibitor) to pantoprazole caused no significant changes in the serum levels of gastrin, PTH, Ca, and ALP compared to the control group. Hence, no significant changes were detected in femoral BMD, femoral bone weight and volume, and trabecular volume. The addition of octreotide (Oct) to pantoprazole also led to no significant changes in the number of osteocytes and osteoclasts. Thus, it seems that pantoprazole could decrease femoral bone densitometry parameters by decreasing the number of osteocytes and increasing the number of osteoclasts, which was secondary to their effects on elevating the serum level of gastrin.

Bone remodeling is a highly controlled, ongoing process that is necessary for maintaining mineral homeostasis and replacing old and damaged bones. Osteoclastic resorption and osteoblastic bone production are closely correlated during the bone remodeling cycle. Osteoporosis is the most frequent metabolic bone disease caused by disruption of the bone remodeling cycle and any ensuing imbalance between bone resorption and production. Osteoblasts are formed by the direct differentiation of mesenchymal stem cells into bone. In connective tissue, mesenchymal stem cells group together and develop into osteoblasts [49]. A type I collagen-rich matrix called osteoid is secreted by mature osteoblasts. An ossification center is formed by the mineralization of the osteoid, from which mineralization spreads. Osteoblasts undergo terminal differentiation to become osteocytes, which are then imprisoned within the developing bone matrix. Early in skeletal development, a process called “bone modelling” affects the size and shape of a bone [50]. The bone must be removed from one anatomical site and replaced at another during this process, which requires the separation of bone resorption and creation. Preserving skeleton morphology during linear growth is a crucial illustration of modelling. The differentiation and function of osteoclasts and osteoblasts are regulated by important osteocyte signaling pathways like the receptor activator of nuclear factor-kB (RANK)/RANK Ligand (RANKL)/osteoprotegerin (OPG) and Wnt, which are also the methods through which numerous hormones finally exercise their effects [51].

Depending on the length of exposure, endocrine modulation of the bone remodeling cycle, including PTH, can have directly conflicting effects on bone remodeling. Continuous PTH promotes bone resorption and plays a crucial role in calcium homeostasis in the body. Additionally, the extended exposure to excessive PTH that develops from parathyroid adenoma or parathyroid hyperplasia in primary hyperparathyroidism causes hypercalcemia, bone loss, and an elevated risk of fracture. Vitamin D 1,25(OH)_2_ controls intestinal calcium and phosphate absorption, supplying the building blocks for bone mineralization. Glucocorticoids, thyroid hormone, and sex hormone (estrogen, androgen) are the main paracrine mechanism growth elements of bone remodeling. Transforming growth factor beta (TGF-*β*), boyevaya mashina pyekhoty 1 (BMPs), prostaglandins, cytokines including IL-1 and IL-6, and tumor necrosis factor (TNF*α*) can all drive osteoclastogenesis, but interleukin 4 (IL-4) and interferon-gamma (INF-*γ*) can decrease osteoclast formation [51, 52]. Many previous studies demonstrated that PPI treatment could reduce BMD [1, 11–13], but few studies failed to find a significant association between PPI use and low BMD or an increased risk of fractures [10, 18, 19, 53, 54]. However, the exact underlying mechanisms have remained unclear. Some possible mechanisms have been discussed here.

First, pantoprazole may decrease calcium absorption from the intestine, thereby enhancing bone resorption [21]. Nonetheless, it has been generally accepted that calcium solubility and absorption are facilitated by gastric acid [55]. Yet, the studies evaluating the impact of hypochlorohydria on calcium absorption have come to similar conclusions [56–58]. Prior research demonstrated a reduction in BMD after partial gastrectomy [59]. However, it might not be necessarily associated with decreased acid secretion. It might result from hypergastrinemia that induces parathyroid hyperplasia, which can promote bone calcium loss [60, 61]. In another investigation, vagotomy without gastrectomy did not increase bone mineral loss. This suggested a limited role for gastric acid in the development of osteoporosis [62]. In the same line, the present study findings revealed no changes in the serum levels of calcium and phosphorous in the pantoprazole rats.

The second mechanism explained for the association between PPIs and osteoporosis is hypergastrinemia secondary to gastric acid suppression by PPIs [63], which can induce parathyroid hyperplasia and increase the PTH level [63]. A prior study showed that persistently high PTH levels led to an increased risk of osteoporosis and fractures [64, 65]. However, Maggio et al. failed to find any significant difference between PPI users and nonusers regarding the PTH serum level [12]. In the present study, the rats in the pantoprazole group had higher serum levels of PTH and gastrin compared to the control and Pan+Oct groups. Moreover, the femoral BMD, BMAD, and bone volume were lower in the Pan group compared to the control and Pan+Oct groups. The results of the stereological study also revealed a decrease in the number of osteocytes and an increase in the number of osteoclast cells in the Pan group compared to the control and Pan+Oct groups. Hence, it was hypothesized that gastrin and PTH played a key role in osteoporosis because adding octreotide to correct hypergastrinemia in the rats consuming pantoprazole caused no significant changes in the number of osteoclasts and osteoblasts compared to the controls.

The third possible mechanism is related to the role of hypomagnesemia secondary to PPI usage [5, 66]. However, similar to the current study, some studies did not find any changes in magnesium serum levels among PPI users [67].

In spite of many interesting findings in the present study that was the first stereological study evaluating the effects of pantoprazole on bone with and without hypergastrinemia, some limitations have to be taken into account. For instance, further studies including bone computed tomography (CT scan) analysis, measurement of serum factors including c-telopeptide of type I collagen (CTX), bone-specific alkaline phosphatase, and gene expression of bone turnover markers are needed to elucidate the exact mechanisms involved in the effects of pantoprazole on bone loss.

## 5. Conclusion

The current study results indicated that three months of treatment of rats with pantoprazole led to an increase in the levels of ALP, PTH, and gastrin. The other effects of pantoprazole were the reduction of bone mineral density parameters as well as femoral stereological indexes. It is worth noting that all unfavorable effects of pantoprazole were inhibited by the addition of octreotide, known as a gastrin secretion inhibitor. Thus, it was hypothesized that the effect of pantoprazole on bone density and bone stereology was caused by the hypergastric state because these adverse effects were not observed in the rats given both octreotide and pantoprazole.

## Figures and Tables

**Table 1:** Serum level of PTH, gastrin, ALP, phosphorous, and Ca levels at baseline before treatment.

Groups	PTH (pg/mL)	Gastrin (ng/L)	ALP (IU/L)	Phosphorus (mg/dL)	Ca (mg/dL)
Control	66.77 ± 3.34	18.58 ± 3.90	230.0 ± 64.9	5.76 ± 0.4	9.62 ± 0.38
Oct	65.37 ± 5.85	19.40 ± 4.20	258.7 ± 26.5	5.70 ± 0.6	9.65 ± 0.31
Pan	63.80 ± 4.91	18.53 ± 1.32	251.3 ± 24.7	5.76 ± 0.7	9.73 ± 0.19
Pan+Oct	65.14 ± 6.85	18.63 ± 2.68	246.2 ± 318	5.87 ± 0.6	9.52 ± 0.29
*p* value	0.679	0.925	0.447	0.940	0.534

Results are presented as mean ± SD (*n* = 10). ^∗^*p* < 0.05 was considered statistically significant.

**Table 2:** Measurement of the baseline of femur bone mineral density (BMD), femur bone mineral content (BMC), and bone mineral apparent density evaluated by a Hologic system Dual-energy X-ray absorptiometry (DXA).

Groups	Femur-BMD (g/cm^2^)	Femur-BMC (g)	Femoral BMAD (g/cm^3^)
Control	0.217 ± 0.02	0.689 ± 0.04	0.068 ± 0.012
Oct	0.213 ± 0.01	0.711 ± 0.04	0.064 ± 0.008
Pan	0.223 ± 0.02	0.710 ± 0.03	0.070 ± 0.012
Pan+Oct	0.216 ± 0.02	0.701 ± 0.04	0.067 ± 0.013
*p* value	0.657	0.569	0.693

Results are presented as mean ± SD (*n* = 10). ^∗^*p* < 0.05 was considered statistically significant.

## Data Availability

The datasets used and analyzed during the current study are available from the corresponding authors on reasonable request.

## Abbreviations

ALP: Alkaline phosphatase
BMC: Bone mineral content
BMD: Bone mineral density
BMAD: Bone mineral apparent density
Ca: Calcium
DXA: Dual X-ray absorptiometry
ECL: Enterochromaffin-like
GERD: Gastroesophageal reflux disease
PPI: Proton pump inhibitors
PTH: Parathyroid-hormone
P: Phosphate.
